# EAACI: A European Declaration on Immunotherapy. Designing the future of allergen specific immunotherapy

**DOI:** 10.1186/2045-7022-2-20

**Published:** 2012-10-30

**Authors:** Moises A Calderon, Pascal Demoly, Roy Gerth van Wijk, Jean Bousquet, Aziz Sheikh, Anthony Frew, Glenis Scadding, Claus Bachert, Hans J Malling, Rudolph Valenta, Beatrice Bilo, Antonio Nieto, Cezmi Akdis, Jocelyne Just, Carmen Vidal, Eva M Varga, Emilio Alvarez-Cuesta, Barbara Bohle, Albrecht Bufe, Walter G Canonica, Victoria Cardona, Ronald Dahl, Alain Didier, Stephen R Durham, Peter Eng, Montserrat Fernandez-Rivas, Lars Jacobsen, Marek Jutel, Jörg Kleine-Tebbe, Ludger Klimek, Jan Lötvall, Carmen Moreno, Ralph Mosges, Antonella Muraro, Bodo Niggemann, Giovanni Pajno, Giovanni Passalacqua, Oliver Pfaar, Sabina Rak, Gianenrico Senna, Gabriela Senti, Erkka Valovirta, Marianne van Hage, Johannes C Virchow, Ulrich Wahn, Nikolaos Papadopoulos

**Affiliations:** 1 Section of Allergy and Clinical Immunology, Imperial College, National Heart and Lung Institute, Dovehouse Street, London, SW3 6LY, UK; 2 Department and INSERM U657, Hôpital Arnaud de Villeneuve, University Hospital of Montpellier, Montpellier, France; 3 Department of Allergology, Erasmus MC-University Medical Center, Rotterdam, The Netherlands; 4 Allergy Department and INSERM U657, Hôpital Arnaud de Villeneuve, University Hospital of Montpellier, Montpellier, France; 5 The University of Edinburgh, Centre for Population Health Sciences, Edinburgh, UK; 6 Department of Respiratory Medicine, Royal Sussex County Hospital, Brighton, UK; 7 The Royal National Throat, Nose and Ear Hospital, London, UK; 8 Upper Airway Research Laboratory (URL), Ghent University Hospital, Ghent, Belgium; 9 Allergy Clinic, National University Hospital, Copenhagen, Denmark; 10 Division of Immunopathology, Department of Pathophysiology and Allergy Research, Centre for Pathophysiology, Infectiology & Immunology, Medical University of Vienna, Vienna, Austria; 11 Department of Internal Medicine, Immunology, Allergy & Respiratory Diseases, University Hospital, Ospedali Riuniti di Ancona, Ancona, Italy; 12 Pediatric Allergy and Pneumology Unit, Children's Hospital La Fe, Valencia, Spain; 13 Swiss Institute of Allergy and Asthma Research (SIAF), University of Zurich, Davos, Switzerland; 14 Centre de l'Asthme et des Allergies, Hôpital d'enfant Armand Trousseau, Paris, France; 15 Department of Allergy, Hospital Clinico Universitario, Santiago de Compostela, Spain; 16 Department of Paediatrics, Respiratory and Allergic Disease Division, Medical University Graz, Graz, Austria; 17 Allergy Division, Ramon & Cajal University Hospital, Alcala de Henares University, Madrid, Spain; 18 Department of Pathophysiology and Allergy Research, Christian Doppler Laboratory for Immunomodulation, Medical University of Vienna, Vienna, Austria; 19 Department of Experimental Pneumology, Ruhr-University, Bochum, Germany; 20 Allergy and Respiratory Diseases Clinic, Department of Internal Medicine, University of Genoa, Genoa, Italy; 21 Allergy Section, Department of Internal Medicine, Hospital Universitari Vall d'Hebron, Barcelona, Spain; 22 Department of Respiratory Diseases, Aarhus University Hospital, Aarhus, C, 8000, Denmark; 23 Service de Pneumologie-Allergologie, Hôpital Larrey, CHU de Toulouse, Toulouse, France; 24 Section of Allergy and Clinical Immunology, National Heart and Lung Institute, Imperial College and Royal Brompton Hospital, London, United Kingdom; 25 Allergy Unit, Children’s Hospital, Kantonsspital Aarau, Switzerland; 26 Hospital Clínico San Carlos, Facultad de Medicina-UCM, IdISSC, Madrid, Spain; 27 Research Centre for Prevention and Health, Glostrup University Hospital, Copenhagen, Denmark; 28 Department of Clinical Immunology, Wroclaw Medical University, Wrocław, Poland; 29 Allergy & Asthma Center Westend, Outpatient Clinic Hanf, Ackermann & Kleine-Tebbe, Berlin, Germany; 30 Center for Rhinology and Allergology, Wiesbaden, Germany; 31 Krefting Research Centre, Department of Internal Medicine and Clinical Nutrition, Institute of Medicine, Sahlgrenska Academy, University of Gothenburg, Gothenburg, Sweden; 32 Seccion de Alergia, Hospital Reina Sofía, Cordoba, Spain; 33 Institute of Medical Statistics, Informatics and Epidemiology (IMSIE), Medical Faculty, University at Cologne, Cologne, Germany; 34 Department of Pediatrics, Padua General Hospital, Padua, Italy; 35 Pediatric Allergology and Pneumology, German Red Cross Clinic Westend, Berlin, Germany; 36 Department of Pediatrics, Allergy Unit, University of Messina, Messina, Italy; 37 Allergy and Respiratory Diseases, University of Genoa, Genoa, Italy; 38 Center for Rhinology and Allergology, Wiesbaden, Germany; 39 Department of Respiratory Medicine and Allergology, Sahlgrenska University Hospital, Goteborg, Sweden; 40 Allergy Service, Verona General Hospital, Verona, Italy; 41 Clinical Trials Center, University Hospital Zurich, Zurich, Switzerland; 42 Suomen Terveystalo Allergy Clinic, Turku, Finland; 43 Division of Respiratory Medicine, Department of Medicine, Karolinska Institutet, Stockholm, Sweden; 44 Department of Pulmonology, Intensive Care Medicine, Zentrum f. Innere Medizin, Klinik I, University Clinic Rostock, Rostock, Germany; 45 Department for Pediatric Pneumology and Immunology, Charité Medical University, Berlin, Germany; 46 UPC Research Laboratories, Allergy Department, 2nd Pediatric Clinic, University of Athens, Athens, Greece

**Keywords:** Allergy, Asthma, Rhinitis, Immunotherapy, Health economics, Quality of life

## Abstract

Allergy today is a public health concern of pandemic proportions, affecting more than 150 million people in Europe alone. In view of epidemiological trends, the European Academy of Allergy and Clinical Immunology (EAACI) predicts that within the next few decades, more than half of the European population may at some point in their lives experience some type of allergy.

Not only do allergic patients suffer from a debilitating disease, with the potential for major impact on their quality of life, career progression, personal development and lifestyle choices, but they also constitute a significant burden on health economics and macroeconomics due to the days of lost productivity and underperformance. Given that allergy triggers, including urbanization, industrialization, pollution and climate change, are not expected to change in the foreseeable future, it is imperative that steps are taken to develop, strengthen and optimize preventive and treatment strategies.

Allergen specific immunotherapy is the only currently available medical intervention that has the potential to affect the natural course of the disease. Years of basic science research, clinical trials, and systematic reviews and meta-analyses have convincingly shown that allergen specific immunotherapy can achieve substantial results for patients, improving the allergic individuals’ quality of life, reducing the long-term costs and burden of allergies, and changing the course of the disease. Allergen specific immunotherapy not only effectively alleviates allergy symptoms, but it has a long-term effect after conclusion of the treatment and can prevent the progression of allergic diseases.

Unfortunately, allergen specific immunotherapy has not yet received adequate attention from European institutions, including research funding bodies, even though this could be a most rewarding field in terms of return on investments, translational value and European integration and, a field in which Europe is recognized as a worldwide leader. Evaluation and surveillance of the full cost of allergic diseases is still lacking and further progress is being stifled by the variety of health systems across Europe. This means that the general population remains unaware of the potential use of allergen specific immunotherapy and its potential benefits.

We call upon Europe’s policy-makers to coordinate actions and improve individual and public health in allergy by:

Promoting awareness of the effectiveness of allergen specific immunotherapy

Updating national healthcare policies to support allergen specific immunotherapy

Prioritising funding for allergen specific immunotherapy research

Monitoring the macroeconomic and health economic parameters of allergy

Reinforcing allergy teaching in medical disciplines and specialties

The effective implementation of the above policies has the potential for a major positive impact on European health and well-being in the next decade.

## Allergy Today: A public health threat of pandemic proportions

At the beginning of the 20^th^ century, allergy was viewed as a rare disease. Since then, several incompletely understood factors have triggered a dramatic increase more evident over the last four decades. Initially, the highest prevalence was in Westernized societies: and current estimates suggest that up to 30% of Europeans could suffer from allergic rhinitis or conjunctivitis, up to 20% of children from asthma, 15% from allergic skin conditions and 8% from food allergy. In other regions of the world, the prevalence, which was previously low, is now increasing [1-3]. The burden peaks in the 20–40 year old age group with clinical symptoms of rhinitis reaching 45%. The worldwide numbers are equally worrying. Almost half a billion people suffer from rhinitis [4,5] and approximately 300 million from asthma [6]. Since many patients do not report their symptoms or are not properly diagnosed, the actual size of the problem could be even larger. Taking into account the upward trends shown by epidemiological studies, EAACI predicts that within the next few decades more than half of the European population will suffer from some type of allergy [7,8].

The problem does not lie simply with respiratory allergies. Food allergies are also becoming more frequent and severe. Occupational allergies, drug allergies and allergic reactions to the venom of stinging insects (which can be potentially fatal) add further complexity and concerns. Finally, new types of allergic diseases and allergies against previously non-allergenic substances are being increasingly reported.

The impact of allergic disease is detrimental both for individual sufferers, their carers and for society as a whole. Patients face impairment in their quality of life, their sleep and mood, their competence at work or school and their overall personal development. Society now confronts increasing associated costs on a scale that will soon become unaffordable. With a current estimate of more than 150 million patients and a prediction of more than 300 million in Europe in the next decade, allergies constitute a public health concern of pandemic proportions requiring immediate action [8-12].

### The impact of allergy on the quality of life of Europeans

At a public health level allergic diseases have a detrimental impact on personal development, career progression and lifestyle choices for patients and their families. People with allergic conditions are at a disadvantage, which affects their school performance, work performance and social life.

Children with allergy demonstrate difficulty in coping at school and develop associated learning difficulties and sleeping problems [13]. As a result, it has been observed that sleepiness and mood swings frequently lead children to be isolated, perform less at school [14] and even get bullied by their peers. Family life and personal relations are subsequently disturbed [15-17].

Adult and adolescent patients also face a significantly higher amount of problems in their workplace due to increased sick days and productivity reduction [18]. Cognitive functions are impaired and can be especially detrimental for school, university or work performance [15,16,19]. Finally, several studies have shown that allergic individuals have a higher risk of developing depression [20] as well as higher risk of depression in mothers with an asthmatic child [21].

The impact of allergies on the quality of life of sufferers can be as high, or higher, than diseases that are perceived as more ‘serious’ (i.e. diabetes). Lately, physicians and scientists have been utilizing a set of specific tools in order to evaluate the different domains of quality of life of allergic patients. The findings stemming from this make us realize the extent of the issues and underline the urgent need for solutions. By focusing on quality of life as a key impact of allergies and asthma, we will be able to give European patients renewed access to optimism. In addition, we should never forget that a small yet significant proportion of allergic reactions might result in death; people at risk need to be prioritized and protected.

### The impact of allergy on health economics and macroeconomics

The associated reduction in productivity and the rising number of sick days taken by allergic patients represents one of the biggest negative impacts on national, business and health economies in Europe [22,23].

Allergy health costs and their continuing escalation have an adverse effect on the European economy due to both direct costs (e.g. in the context of considering asthma alone, pharmaceutical costs stands at €3.6 billion per year and health care services at a further €4.3 billion per year) [24] and, perhaps even greater, indirect costs. In total, 15% of the population is receiving long-term treatment in Europe for allergies and/or asthma, making it the most common reason for a long-term treatment in children and young people [25]. Among the direct medical costs, the primary components are diagnostic tests, consultations and medication, while an additional major cost item is hospitalization, usually associated with severe exacerbations of asthma or severe anaphylactic reactions [25,26].

Moreover, performance deficits, loss of productivity and absenteeism are closely linked to allergy suffering and have a major effect on macroeconomics. Asthma and rhinitis are estimated to result in more than 100 million lost workdays and missed school days each year in Europe (not only children absent from school on any given day, but also parents' productivity or absence from work) [27-29].

Recently, it became apparent that in addition to absenteeism, hundreds of millions of Euros are also lost by “presenteeism”, a condition in which people go to work, but are unable to perform to their optimum capacity. The total cost of asthma alone is estimated at more than €25 billion annually [24]. The cost of rhinitis is probably higher but, unfortunately, large scale socioeconomic studies in Europe are lacking. Unpublished investigations by the European allergy network GA^2^LEN calculate the current loss due to untreated allergic rhinitis-related presenteeism to be approximately a €100 billion annually to employers [30]. This is based on employment figures from European statistics, but does not measure the loss to society due to presenteeism at schools or universities. Understanding and monitoring the costs of allergic diseases should be a priority and all health care systems should take into account the rapid increase in prevalence, increase in severity and cost of allergies as they may receive unsustainable demands from these conditions alone.

### The unsustainability of allergy’s current symptomatic treatments

Currently, allergies are in most cases treated by short-term symptom relieving or long-term anti-inflammatory drugs [31-33]. The introduction of the latter, of which corticosteroids are the most prominent, has reduced some of the more serious outcomes of these diseases [34]. However, important drawbacks in regard to pharmacotherapy have also become evident: firstly, the effectiveness of current medications in controlling allergy symptoms is suboptimal [35]. Even under the well-controlled conditions of a clinical trial, and after optimizing treatment, a considerable proportion of patients, sometimes even higher than 50%, will continue to experience troublesome symptoms [35]. Secondly, and most importantly, even after years of a continuous, effective treatment, symptoms relapse very shortly after ceasing daily use of medication [31]. Finally, long-term use of drug treatment increases the possibility, but also the fear, of adverse effects [36,37]. This is unacceptable for patients who respond with a characteristic lack of compliance to medical advice and frequently resort to non-proven – and often expensive – complementary and alternative ‘treatments’ with poor results and which even may, if anything, exacerbate the problem [38].

With the increasing costs of newer medications and increasing number of sufferers, this continuous dependence on drugs is unsustainable. Both patients and physicians call for more effective symptom control, but also for treatments with long-term effects: a cure of the disease is what should be the target for researchers and public health decision makers in the coming years.

### The promise for a cure and the role of allergen specific immunotherapy

Aspects of modern-day European lifestyle, including dietary patterns, urban living, industrialization, exposure to cigarette smoke and other pollutants, and several other factors, are major triggers of symptoms in allergic patients and these are not expected to change on a significant scale within the next few years. It is therefore important to strengthen and optimize preventive and treatment strategies. This has been clearly stated in the EU Sustainable Development Strategy; all European citizens should have the means to improve their quality of life, and mental and physical health, and have access to the best preventive measures [39].

Allergen specific immunotherapy with preparations which have confirmed effectiveness and tolerability in adequate studies covering the specific claims made, is effective in alleviating allergy symptoms to a similar (or even larger) extent than pharmacological treatments for asthma [40], allergic rhinitis [41,42] and allergic conjunctivitis [43]. At present it is the only curative treatment for *Hymenoptera* (bee and wasp) venom allergy. Unlike symptomatic medications, the benefits of allergen specific immunotherapy continue several years after discontinuation of the treatment [44-46]. Moreover, specific products for allergen specific immunotherapy have shown to have disease-modifying capacities being able to prevent the progression of allergic diseases, as in the case of hay fever that may frequently lead to asthma [47-51] and to reduce the risk of new sensitizations. Recent studies have shown that allergen specific immunotherapy has a role in the treatment of food allergy in order to reduce the risk of fatal anaphylaxis. Therefore, allergen specific immunotherapy is currently the only medical intervention that could potentially reverse the increasing disease trends being seen in Europe and elsewhere.

Allergen specific immunotherapy has been used as a medical treatment for over a century [52], offered mostly to the more severe or difficult patients, in whom use of medications is unsatisfactory, either because of lack of efficacy or because of unacceptable untoward effects. Currently, it is used only as a second-line treatment [53]. However, in recent international guidelines and academic position statements, it has been advocated for use in those with milder disease in order to prevent chronic irreversible structural changes in the airways. Allergen specific immunotherapy should thus be considered as a treatment strategy in those with early-onset and/or mild disease in order to maximise the potential for the all-important disease-modifying capacities [4,53].

Major technological advances in the quality and formulation of extracts used, new and more patient-friendly delivery systems and a deeper understanding of the mechanisms of allergic diseases have all led to the expectation of a major breakthrough in allergy treatment, in which allergen specific immunotherapy should play a crucial role.

### What allergen specific immunotherapy can achieve

Allergen specific immunotherapy holds considerable promise for patients and, by extension, for society as a whole. When used properly, following careful diagnosis, and with good quality, well-characterized and clinically documented extracts, it can transform the life of people living with allergic diseases.

#### For patients

Allergen specific immunotherapy has been found to be effective in reducing symptoms of allergic rhinitis and allergic asthma and improving the quality of life of allergy sufferers. It also results in reduced use of symptom relieving medications. Allergen specific immunotherapy has the potential for important longer-term benefits, even after cessation of the treatment. In patients with allergy to insect venom or food allergy, allergen specific immunotherapy is able to prevent life-threatening reactions. Different routes for allergen specific immunotherapy have been evaluated, such as the subcutaneous, sublingual, oral, nasal, bronchial, and intra-lymphatic, the first two of these routes being the most commonly used in clinical practice today. The most common allergens used in clinical practice are aero-allergens for seasonal and perennial allergy; more recently, latex and food allergens have been evaluated in clinical trial with promising results to be confirmed [54].

#### For clinicians

Both allergy specialists and general internists/family physicians benefit from a therapeutic intervention that not only reduces symptoms in their patients, but also gives strong hope that the underlying allergy will be cured and/or stopped in its progression. Especially in children in whom the prospect of one allergy following the other (the ‘allergic march’) is ever present, it also offers a way for putting a break on this process, by preventing the progression from, for example, allergic rhinitis to asthma.

#### For public health

Allergen specific immunotherapy is currently the only treatment that offers the possibility of reducing long-term allergic disease burden and thereby the considerable costs associated with treatment; this is achieved by beneficially altering the natural course of the disease. Several pharmaco-economic studies have shown important benefits even from early time points, with steady increase with time. It is conceivable that further research may lead to preventive vaccination for allergies, as is now well-established in relation to many infectious diseases.

### Long-term effects of allergen specific immunotherapy

There is encouraging emerging evidence that children receiving allergen specific immunotherapy for allergic rhinitis develop considerably less asthma 10 years later, in comparison to children that do not (control) [47], supporting the case for allergen specific immunotherapy in preventing progression of allergies to more severe forms. Given the considerable potential associated with this immune-modulatory treatment approach, this is an area in which there is a need for considerably more research.

### Major milestones for allergen specific immunotherapy

It has taken considerable time to reach the current degree of effectiveness and safety of allergen specific immunotherapy. Several appropriately designed clinical trials have proven the effectiveness of allergen specific immunotherapy of specific products in allergic rhinitis, asthma and venom allergy. Such trials have not been easy to design and perform for many reasons: extracts, populations, dosing schedules, disease localization, and allergen exposure are among the factors that vary considerably and have to be taken into account [55-57]. Nevertheless, different independent systematic reviews and meta-analyses of blinded randomized controlled studies have consistently confirmed efficacy and effectiveness; with the recent large phase III trials, allergen specific immunotherapy has moved from experience and dogma to evidence based medicine and facts [58,59]. Furthermore, long-term effects have been repeatedly shown, after treatment cessation. Based on these findings, national and international evidence-based guidelines have been developed in order to assist practising physicians in selecting the appropriate patients and preparations and in optimizing treatment.

To this end, the continuous improvement of technologies that lead to high quality extracts and formulations have had a major beneficial impact on both safety and efficacy of allergen specific immunotherapy. Moreover, the new delivery routes, such as sublingual allergen immunotherapy, have further added to the armamentarium of allergy specialists, offering more convenient solutions and high safety.

Molecular allergology is expected to take the field to the next step, as the components of treatment will be defined to precision in quality and quantity.

### Major bottlenecks for further diffusion of allergen specific immunotherapy

Extensive further research is needed to maximize the potential of allergen specific immunotherapy:

Even small changes in dose schedules may affect results both in efficacy and safety. The potential schemes are numerous and should be examined comprehensively.

The design of the studies, their analysis and how to interpret their results should be refined bearing in mind that pharmacotherapy and immunotherapy of allergic diseases have commonalties and differences.

Although we are much closer than ever to understanding the basic mechanisms of allergen specific immunotherapy, there are still several open questions which, if answered, would enable us to manipulate immune responses that are already established.

New extract preparations and especially vaccines containing molecular components require validation. The complexity of possible component combinations requires novel bioinformatic approaches. Studies exploring cost-effectiveness of allergic rhinitis and asthma are still lacking and should be assessed in relation to the various health systems across Europe. The macro-economic impact of allergies and the long-term cost-effectiveness of allergen specific immunotherapy need further detailed evaluation and attention.

Although it represents one of the most rewarding fields in terms of return on investments, translational value and European integration, allergen specific immunotherapy has not received adequate attention from European research funding bodies. It is a field in which Europe is already recognized as a leader worldwide and it is important that we now capitalize on this leadership position.

Awareness of allergen specific immunotherapy and its treatment potential is inadequate in the general population. In some cases immunotherapeutic approaches are mistakenly considered to be ‘alternative’, non-proven treatments.

### Call for action

Allergic diseases, including asthma, are amongst the top smoldering risks of global healthcare. The need to deploy effective treatment solutions such as allergen specific immunotherapy to stop and potentially reverse allergy’s impact on European health, well-being and macroeconomics is more urgent than ever before.

We call upon Europe’s policy makers to co-ordinate actions and improve individual and public health in allergy by:

### Promoting allergen specific immunotherapy awareness

The pandemic dimensions of allergic diseases highlights the need for awareness at all levels. Millions of patients who see little or no improvement with symptomatic drug treatments, or wish to adopt a more curative approach to their illness can benefit from allergen specific immunotherapy and should therefore be aware of the availability and benefits of such treatment. Awareness campaigns, and patient educational programs at pan-European or national levels, should be promoted in order to maximize the effects of the treatment on Europe’s population.

### Update national healthcare policies to support allergen specific immunotherapy

The huge socio-economic burden of allergic disease calls for setting priorities. By prioritizing allergen specific immunotherapy in health planning and by designing healthcare policies that support allergen specific immunotherapy treatments of allergy through national health insurance subsidization, long-term effects of allergic diseases at a national, social and individual level will be substantially reduced. The prevention of allergic diseases can result not only in significant cost reduction, but also in a major improvement of the quality of life of Europeans.

### Prioritize funding for allergen specific immunotherapy research

There has in recent years been tremendous progress in effectively diagnosing and treating specific allergies. Treatment approaches can and should be optimized. Innovative approaches are underway. Recent advances in molecular technology are destined to revolutionize immunotherapy treatments. Allergen specific immunotherapy research needs to be catalyzed by European research funding schemes, as the majority of current funding derives from the industry, thus focusing only on part of the treatment’s full capacity.

### Monitoring the macroeconomic and health economic parameters of allergy

There is a need for cost-benefit, cost-effectiveness and cost-utility analyses as allergic diseases are increasingly affecting large numbers of people with substantial cost implications. This is an important part of the need to monitor allergies in general, taking into account the rapid changes in prevalence and their widespread implications. Treatments like allergen specific immunotherapy that can combat both the symptoms and the long-term consequences can be more cost effective than routine health care by breaking the vicious circle of living with allergies and coping with prolonged periods of suffering and medical treatment^.^

### Streamline medical disciplines and specialties

Health systems around Europe differ widely in regard to the provided services and range of health care professionals who address allergies. This results in insufficient and unequal access to allergy services. Allergen specific immunotherapy is a highly specialized value-added treatment that can only be delivered by allergy specialists. However, the enormous number of allergic patients requires a wide range of health care professionals to be constantly trained and informed of new evidence as well as being equipped with appropriate tools to adequately respond to the expanding allergy incidents and patients’ needs.

The effective implementation of the above policies would have a major positive impact in European health and well-being in the years to come.

## Competing interests

All authors are or have been EAACI officers, are prescribers of allergen immunotherapy, have participated in immunotherapy research and/or have received honoraria from immunotherapy manufacturers for participation in trials, as speakers or advisors.
